# The Perfect Match: Assessment of Sample Collection Efficiency for Immunological and Molecular Findings in Different Types of Fabrics

**DOI:** 10.3390/ijms231810686

**Published:** 2022-09-14

**Authors:** Sara C. Zapico, Alex Dytso, Leticia Rubio, Gabriela Roca

**Affiliations:** 1 New Jersey Institute of Technology, Department of Chemistry and Environmental Science, 161 Warren Street, Tiernan Hall, 365, Newark, NJ 07102, USA; 2 Smithsonian Institution, National Museum of Natural History, Anthropology Department, 10th and Constitution Ave., NW, Washington, DC 20560, USA; 3 New Jersey Institute of Technology, Department of Electrical and Computer Engineering, University Heights, Newark, NJ 07102, USA; 4 Fulbright Visiting Scholar Program, Department of Chemistry and Environmental Science, 161 Warren Street, Tiernan Hall, 365, Newark, NJ 07102, USA or; 5 Department of Human Anatomy and Legal Medicine, Facultad de Medicina, Universidad de Málaga, 29071 Málaga, Spain; 6 SERATEC®, Gesellschaft für Biotechnologie mbH, Ernst-Ruhstrat-Strasse 5, 37079 Göttingen, Germany

**Keywords:** saliva, immunochromatographic tests, cotton swabs, nylon flocked swabs, STR profile, mtDNA

## Abstract

Body fluid identification at crime scenes can be crucial in retrieving the appropriate evidence that leads to the perpetrator and, in some cases, the victim. For this purpose, immunochromatographic tests are simple, fast and suitable for crime scenes. The potential sample is retrieved with a swab, normally a cotton swab, moistened in a specific buffer. Nonetheless, there are other swab types available, which have been proven to be efficient for DNA isolation and analysis. The aim of this study is to evaluate the efficiency of different swab types for body fluid identification as well as DNA isolation and characterization. Fifty microliters of human saliva were deposited in three different types of fabric (denim, cotton, and polyester). After 24 h at room temperature, samples were recovered by applying three different swab types, and the tests were performed. Subsequently, total DNA was recovered from the sample buffer. Cotton swabs performed worse in denim and cotton fabrics in both immunochromatography tests and DNA yield. No differences were observed for polyester. In contrast, and except for two replicates, it was possible to obtain a full DNA profile per fabric and swab type, and to identify the mtDNA haplogroup. In this paper, the impact of swab types on body fluid identification through the application of immunochromatographic tests is analyzed for the first time. This work corroborates previous research related to the influence of swab types in nuclear DNA isolation and characterization.

## 1. Introduction

The identification of body fluids at crime scenes or in the lab is important for correct evidence handling, but may also help with crime reconstruction [1]. Additionally, it allows for further testing, including DNA analysis [2]. 

Body fluids commonly found at crime scenes are blood, vaginal secretions, semen, urine and saliva. The detection of saliva at crime scenes can be carried out through different methods. One of them is the alternative light sources (ALSs); in particular, saliva can be detected at a wavelength range of 415-490 nm [3]. Further tests for saliva identification include amylase detection. Although α-amylase is present in all body fluids, the α-amylase activity of saliva is much higher than that of other body fluids [4,5,6]. There are different methods for identifying α-amylase based on chemical reactions [2]; however, the most common and quickest tests are the lateral flow immunochromatography (LFI) tests based on antigen–antibody reactions. These tests detect the presence of α-amylase, not its activity. These tests use a mobile and stationary monoclonal anti-human salivary α-amylase antibody that forms a visible pink line in the presence of this protein [3,7]. There are some studies aiming to improve saliva detection through new methodologies such as Raman spectroscopy [8] or the detection of salivary bacteria [9]; however, there are no studies focusing on analyzing the different factors that could affect saliva detection with these LFIs, which are widely used by crime scene investigators and forensic scientists. 

From another perspective, and as described above, the second step after body fluid identification is to obtain the DNA profiling from the sample. This could be carried out using the swab used previously for the LFI or from another sample [10]. Cotton swabs have traditionally been used for DNA retrieval at crime scenes [11]; nonetheless, other swabs are available, such as nylon flocked swabs. Current research focuses on the assessment and comparison of these swabs for DNA retrieval and profiling [12,13,14,15,16,17,18]. Despite these studies, there is no research analyzing the impact of the swab type on body fluid identification with LFI. This could also have implications for other applications of these LFIs, e.g., current COVID-19 rapid tests are LFIs. These tests have been developed so quickly for their purpose that the impact of different factors, for instance, the swab used for sample collection, has not been analyzed yet [19,20]. Although the present article is focused on forensic tests, the basic principle is the same as current COVID-19 rapid tests; thus, the findings from this research could have ramifications and promote further research to improve COVID-19 testing. 

Thus, the aim of this study is to assess the efficiency of different swab types for body fluid identification based on immunological approaches with these LFIs, as well as DNA isolation and characterization. 

## 2. Results

### 2.1. Immunochromatographic Tests Results

**Figure 1:**
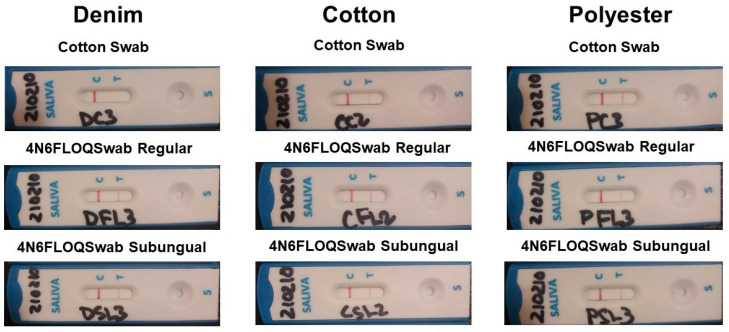
Representative results of immunochromatographic tests per fabric and swab type. All results are positive in these images. As examples of scale values, the cotton swab in denim fabric in the first line shows a weaker band G3.5; in the same fabric, Regular FLOQ swabs show a G8 intensity as well as the subungual swab.

**Figure 2:**
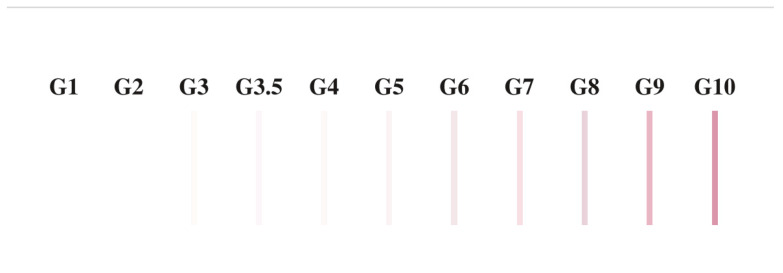
Scale to assign rank values to the immunochromatographic test signal intensity. G3.0 and over are considered positive values to the presence of the protein in question. This scale is used for comparative purposes and analyses only in a fixed experimental design. It was used and tested previously as Internal Quality control from SERATEC^©^ (personal communication).

A band indicates the presence of the protein, and as a result, a positive immunochromatography test (Table 1). This experiment showed differences among swab types. In denim and cotton, band intensities were higher with 4N6FLOQSwabs™ Regular-size tip and 4N6FLOQSwabs™ Subungual Shape than with cotton swabs. In contrast, polyester showed the same intensities in the three swab types. The application of a Kruskal–Wallis test confirmed the significant of the differences among the three swabs in denim (*p* = 0.48) and cotton (*p* = 0.43), but not in polyester (*p* = 0.219). Figure 1 shows a representative immunochromatography test per fabric and swab, and Table 1 shows the ranks assigned to each test based on the scale in Figure 2.

### 2.2. DNA Quantification

Qubit assessment of DNA concentration showed the same pattern, obtaining higher yields of DNA with 4N6FLOQSwabs™ Regular-size tip and 4N6FLOQSwabs™ Subungual Shape in denim and cotton, and similar concentrations with the three types of the swab in polyester. Table 1 shows the results of DNA quantification. 

The Cramer–Von Mises test for normality reported a *p*-value of 0.8862 (*p* close to one indicates normality). After checking the hypotheses of normality and variance homogeneity in the three types of fabric, a one-way ANOVA confirmed that there were statistically significant differences among the DNA concentrations based on swab types in denim (*p* = 0.006) and cotton (*p* = 0.001), but not obtaining significant differences in polyester (*p* = 0.909). The power of the ANOVA for the considered sample size and the significance level of 0.05 was computed to be 0.7698 for denim, 0.9841 for cotton, and 0.05 for polyester, respectively. Note that the power of polyester is low and consistent with the fact that no significant difference was observed there. Thus, the *p*-values, together with the power of the test, indicate statistically significant findings for denim and cotton. 

After that, a two-way ANOVA was carried out to assess the influence of both clothing and swab types on DNA yield. The ANOVA demonstrated that, effectively, both factors play a key role in DNA concentration (swab type F = 14, *p* = 0; fabric F = 6.304, *p* = 0.008; swab and fabric F = 9.193, *p* = 0). 

Specific human quantification with the PowerQuant System ratified these previous results, obtaining higher yields of DNA with 4N6FLOQSwabs™ Regular-size tip and 4N6FLOQSwabs™ Subungual Shape in denim and cotton, and similar concentrations with the three types of the swab in polyester. Table 1 shows the results of human DNA quantification. Additionally, the statistical analyses of these results confirmed previous findings with the Qubit quantification, obtaining significant differences among swab types applying one-way ANOVA in denim (*p* = 0.005) and cotton (*p* = 0.001), but not polyester (*p* = 0.453). Two-way ANOVA confirmed that both fabric and swab types have influence on DNA yield (swab type F = 17.576, *p* = 0; fabric F = 8.525, *p* = 0.002; swab and fabric F = 8.124, *p* = 0.001). 

**Figure 3:**
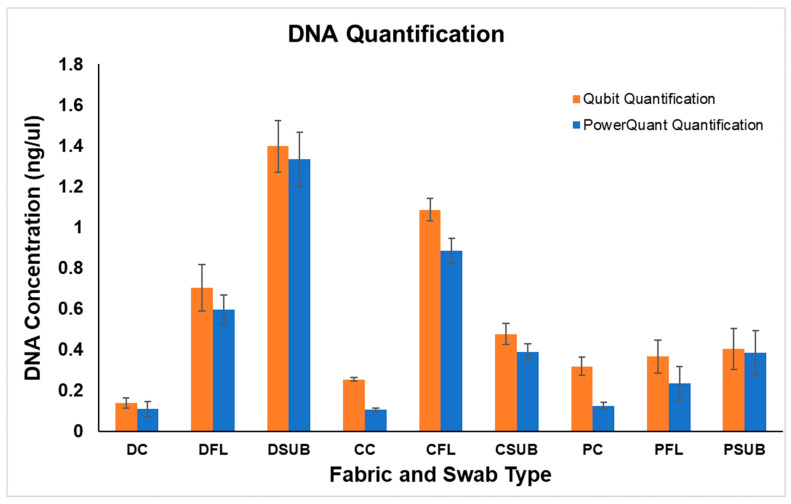
Graphical comparison between Qubit Quantification vs. PowerQuant Quantification, including standard error bars. DC, Denim Cotton Swab; DFL, Denim Flock Swab; DSUB, Denim Subungal Swab; CC, Cotton Cotton Swab; CFL, Cotton Flock Swab; CSUB, Cotton Subungal Swab; PC, Polyester Cotton Swab; PFL, Polyester Flock Swab; PSUB, Polyester Subungal Swab.

There are some inconsistences between the DNA amount measured by Qubit versus the PowerQuant System. It must be considered that the approach of both systems is different. Qubit is based on the intercalation of a fluorescent dye on the DNA, while PowerQuant is based on the amplification of specific human genes. Figure 3 shows a graphical comparison between Qubit quantification and PowerQuant quantification, including the standard errors. The results of both systems, supported by the statistical analyses, confirmed our findings and also correlated with the outcomes of the LFI, as described in the previous section. Table 1 depicts the comparison of LFI test results with Qubit DNA Quantification and PowerQuant Quantification.

### 2.3. DNA Profiling

**Figure 4:**
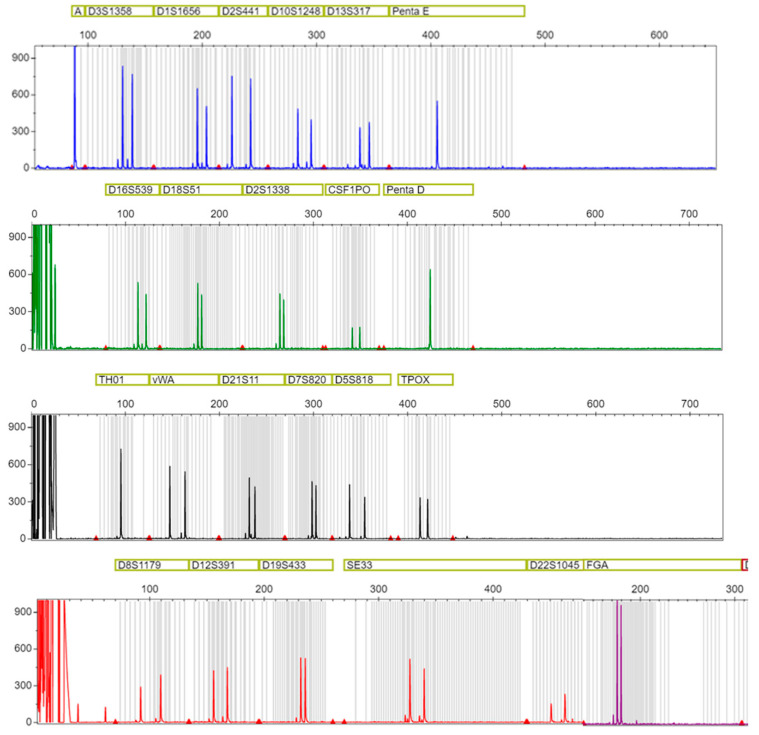
Representative STR profile obtained from the samples. The Y-axis represents relative fluorescent units (RFUs); the X-axis represents fragment lengths in base pairs. On the top, the STR is analyzed. The peaks represent fragments of the respective STR locus. Two peaks represent a heterozygous, one peak represent a homozygous. A represents Amelogenin, which determines the sex. In this case, the donor carried the X,X chromosomes, thus, the sample came from a female. Please note that FGA has a different color as it is identified by a different component; however, for representation purposes, it has been combined with the last row of the profile. Allele calls were removed for privacy.

Except for one replicate of denim fabric with a cotton swab, and one replicate of cotton fabric with a cotton swab, it was possible to obtain a full DNA profile per fabric and swab type with 1 ng of DNA based on Qubit quantification. An example of the full DNA profile is shown in Figure 4. 

**Figure 5:**
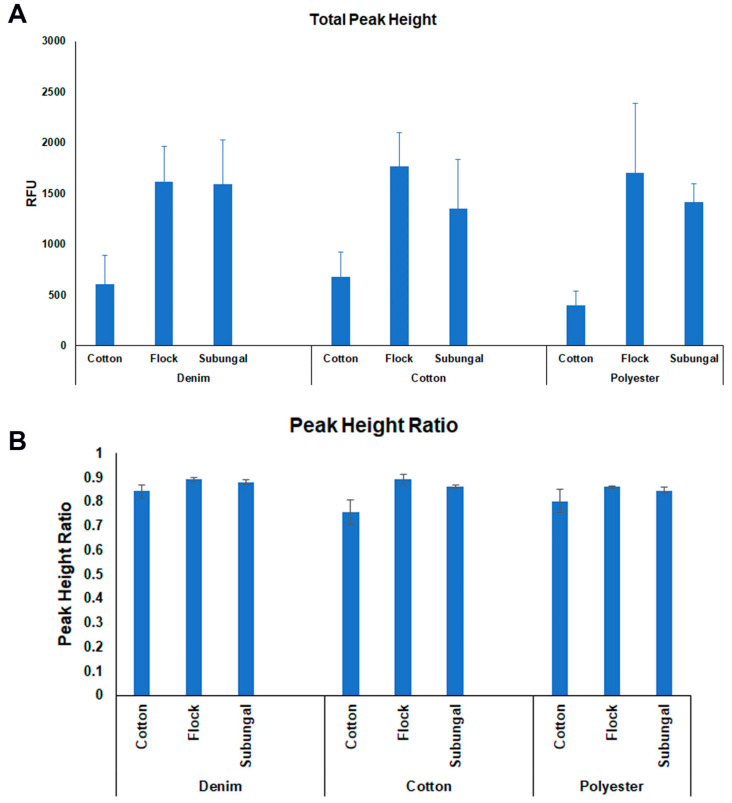
Average of Total Peak Height (TPH) (**A**) and Peak Height Ratio (PHR) (**B**) of the three independent experiments of each fabric and type of swab based on the results of 24 STRs. Total Peak Height (TPH) is represented in Relative Fluorescent Units (RFUs).

In order to assess the quality of the profile, three parameters were calculated based on previous publications [21,22,23]: total peak height, peak height ratio, and calculation of Interlocus Balance. All metrics for these calculations are available upon request. Boxplots depicting these calculations are included in Supplementary Materials. Figure 5 represents the average of total peak height (TPH) in RFU (A) and the average of peak height ratio (PHR) of the STRs (B) in the three independent experiments of each category. 

After checking the hypotheses of normality and variance homogeneity, a two-way ANOVA was carried out to assess the influence of fabric and swab type on these three parameters, and as a result, on the quality of DNA; however, only the type of swab seems to have an impact on DNA quality on total peak height (swab type F = 7.352, *p* = 0.005; fabric F = 0.062, *p* = 0.940; swab and fabric F = 0.113, *p* = 0.976); peak height ratio (swab type F = 7.382, *p* = 0.005; fabric F = 1.672, *p* = 0.216; swab and fabric F = 0.866, *p* = 0.503), not finding an impact of any of these factors on interlocus balance (swab type F = 0.649, *p* = 0.534; fabric F = 0.774, *p* = 0.476; swab and fabric F = 0.649, *p* = 0.534).

### 2.4. mtDNA Sequencing

**Figure 6:**
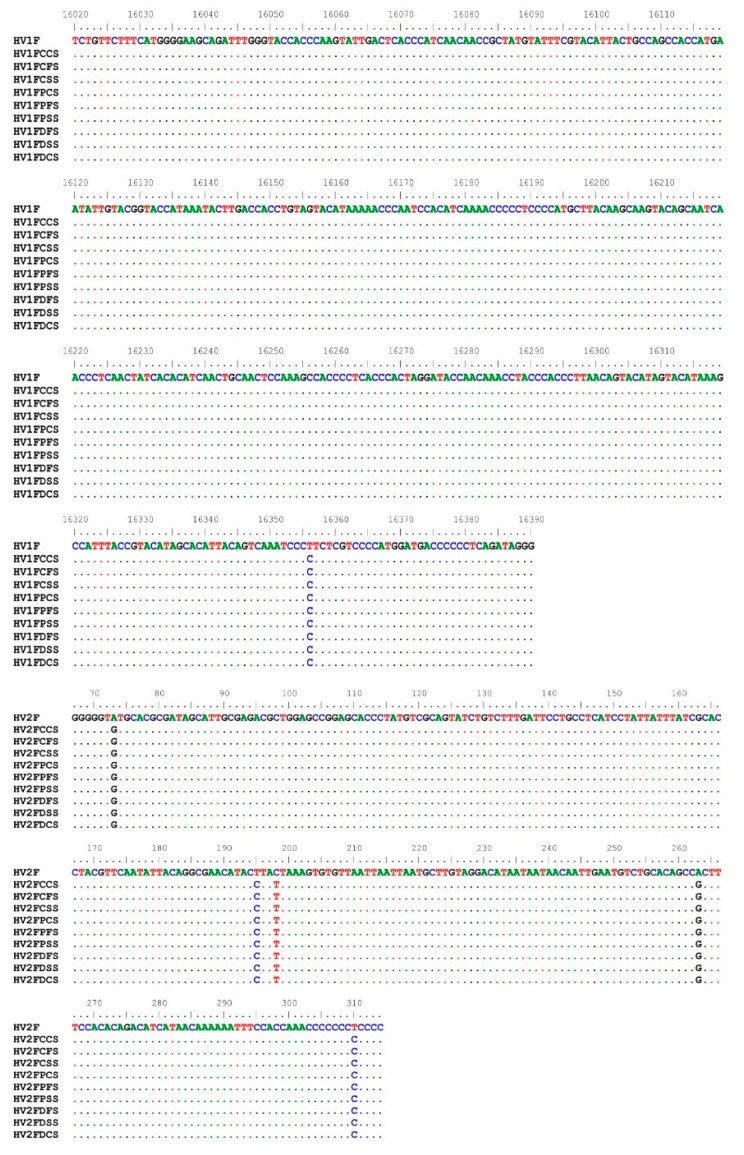
Representative HV1 and HV2 regions of mtDNA showing the mutations on the samples with respect to the revised Cambridge Reference sequence (in the top). CCS, Cotton Cotton swab; CFS, Cotton Flock swab; CSS, Cotton Subungal swab; PCS, Polyester Cotton swab; PFS, Polyester Flock swab; PSS, Polyester Subungal swab; DCS, Denim Cotton swab; DFS, Denim Flock swab; DSS, Denim Subungal swab. All samples analyzed yielded the donor’s haplogroup.

It was possible to sequence the mtDNA on HV1 and HV2 in all types of fabrics and swabs, determining mutations. Based on those mutations, the mtDNA haplogroup was U4 according to EMPOP database in all the samples. Negative controls did not retrieve any quantifiable DNA, and as a result, mtDNA was not assessed in these samples. A representative mtDNA profile of these regions with each sample type is depicted in Figure 6.

## 3. Discussion

Current research on body fluid identification focuses on improving this task by applying new cutting-edge techniques such as FT-IR [24,25]; Raman spectroscopy [8,26,27,28]; or, in the case of saliva, through species identification [9]. Immunochromatographic tests are widely used and accepted by the forensic science community and law enforcement agencies as presumptive and sometimes confirmatory tests both in the field and in the lab towards the identification of the fluid. 

Present efforts should focus on improving current techniques and studying the factors that could hamper the correct identification of body fluids. Additionally, these LFIs are also used for other applications, e.g., current COVID-19 rapid testing, where swabbing is required to retrieve the sample. Thus, based on its wide application, it is important to assess the impact of swab type on sample detection and identification. 

Forensic laboratories are using saliva tests as presumptive tests for identification, assessing α-amylase, which is found in all body fluids, but in higher amounts in saliva, also a source of human DNA. This paper is the first to analyze the influence of swab type on body fluid identification by applying immunochromatographic tests. As described in the results and supported by statistical analysis, in denim and cotton fabrics, saliva detection was weaker when the samples were recovered with cotton swabs; higher intensities with 4N6FLOQSwabs™ Regular-size tip and 4N6FLOQSwabs™ Subungual Shape were found, and no differences among swabs in polyester were observed. Since the next step in the forensic workflow would be DNA isolation and characterization, the present paper also analyzes the impact of the swab on DNA quantification. These results matched the immunochromatographic test results; DNA yield on denim and cotton fabrics was lower with cotton swabs; in contrast, there were no differences among swabs in polyester. According to two-way ANOVA, both clothing and swab type influence the DNA yield. Regarding the quality of nuclear DNA profile, based on these three parameters, it appears that the quality of DNA profile in terms of total peak height and peak height ratio is influenced solely by the type of swab. However, as our findings have proven, independently of the immunochromatographic test results, it was possible to obtain a full DNA profile, per fabric and swab type, indicating that the workflow for body fluid identification is also a valuable tool for the end objective: the identification of the perpetrator and/or victim. Along these lines, differences in the amount of DNA, represented in Table 1, were not significant at the time the specimens were identified with the DNA profile. 

There are no studies in the literature analyzing the influence of swab type in body fluid identification, which makes this work essential in this field and which could be translated to other applications, such as the aforementioned COVID-19 LFI tests, promoting more studies in this area [29]. There are several works studying the impact of different swab types on DNA yield and profiling, as the final aim of a forensic investigator is to identify the perpetrator, or, in some cases, the victim, through DNA analyses. The majority indicated that 4N6FLOQSwabs are the best ones for this purpose, with respect to cotton swabs [17,18]. Cotton swabs have a mattress design, cotton is tightly wound around the wooden/plastic shaft to make the bud [30]; in contrast, flocked swabs are made of parallel short nylon strands flocked onto a plastic stick, and the absorption of the samples is accomplished by capillary action via the nylon fiber [17]. Based on the aforementioned designs, it is claimed that the latter is more effective at releasing cellular materials than cotton swabs. Despite these statements and previous works, other studies denoted that swab efficiency depends on biological material type, substrate, and extraction method [11,13]. Thus, other works focus on improving DNA extraction from cotton swabs or other swabs, applying different modifications to the DNA extraction protocol [31,32,33], or performing a direct PCR [15]. The present study demonstrates how the substrate type, in this case, the fabric, could also be a factor in both body fluid identification and DNA quantification, without affecting the DNA profile quality. Sherier et al. [16] assessed the efficiency of microFLOQ swabs in cotton fabrics for blood, saliva, and semen, obtaining better DNA profiles than with cotton swabs. 

Cotton swabs are less efficient in some circumstances, as depicted in our results. The common knowledge of cotton swabs being less efficient for forensic use must be considered together with other factors such as storage, costs, and waste generation. This information would be essential for forensic labs, police departments or hospitals, in case of COVID-19 tests, to determine whether the extra costs associated with non-standard (cotton) swabs are worthy, depending on the sample. In the case of difficult samples or undefined difficult textiles, it might be better to use nylon swabs, but not for routine tests.

A broader evaluation, including more body fluid assessment types and evaluating results over longer sample incubation periods on the substrate, is part of a separate study. Twenty-four hours indoors at room temperature is already relevant for laboratory testing, and as a proof-of-concept of extensive work. 

## 4. Material and Methods

### 4.1. Saliva Samples, Fabrics, and Swabs

Saliva was retrieved from one donor by spitting in an Eppendorf tube. The New Jersey Institute of Technology Institutional Review Board (IRB) approved the procedures related to human body fluid experimentation (protocol number: 2110013076). Three different types of fabrics were used for this experiment: denim, cotton, and polyester. Three different swab types were assessed: cotton swabs (cotton-tipped applicators sterile, wood shaft, SARSTEDT, Nümbrecht, Germany), Copan 4N6FLOQSwabs™ Regular-size tip, and Copan 4N6FLOQ Swabs™ Subungual Shape (COPAN Italia, Brescia, Italy)—both Copan swabs are composed of nylon fibers arranged in a perpendicular fashion. 

### 4.2. Experiment

Fifty microliters of saliva were deposited on the fabrics (denim, cotton, and polyester). Three replicates per swab type and fabric were evaluated. The size of the sample was used based on the most popular type of clothes and available swabs, where the physical characteristics between them are very clear. After 24 h at room temperature, saliva samples were recovered by applying three different swab types. The swab was moistened in the extraction buffer provided with the SERATEC^®^ SALIVA CS (SERATEC^®^, Göttingen, Germany) test. Then, the swab was applied to the sample in a circular motion for approximately 30 s. Swabs were incubated in agitation in 300 µL of extraction buffer for 10 min. After that, three drops of the buffer were added to the SERATEC^®^ SALIVA CS immunochromatographic tests, and the results were recorded. Negative controls were carried out by swabbing, with each swab type, a part of the fabric without a sample. Positive controls were performed by using a direct saliva sample on the test. In order to assess the results of immunochromatographic tests, the color scale shown in Figure 2 was used. This scale was used in order to compare the samples among themselves and not to quantify them. This type of scale is used by SERATEC^®^ as an internal control to assess the results of the tests.

### 4.3. DNA Extraction

Total DNA was isolated from the swab extraction buffer using a modification of the DNeasy Blood and Tissue Kit (Qiagen^®^). Because part of the buffer from the previous test was used in test performance, more SERATEC^®^ SALIVA CS test extraction buffer was added to the Eppendorf tube up to 400 µL. Then, the swab was kept in the Eppendorf tube, and 20 µL of Proteinase K and 400 µL of Reagent AL were added. The mixture was vortexed for 15 s and incubated in agitation at 56 °C for 10 min. After that, 400 µL of Ethanol was added and vortexed for 15 s. The mixture was transferred to the column following the manufacturer’s protocol. DNA was recovered in 50 µL of buffer AE. 

### 4.4. DNA Quantification

DNA quantification was performed using the Qubit dsDNA Assay Kit (Life Technologies, Carlsbad, CA, USA) along with the Qubit Fluorometer 3.0, according to the manufacturer’s protocol and previously published protocol [34]. 

Specific human DNA quantification was carried out using the PowerQuant System (Promega Corporation, Madison, WI, USA) along with the QuantStudio (Thermo Fisher Scientific, Waltham, MA, USA), according to the manufacturer’s protocol. 

### 4.5. Nuclear DNA Profiling

Promega PowerPlex^®^ Fusion 6C System (Promega Corporation, Madison WI, USA) was used to amplify and characterize 23 autosomal STRs and Amelogenin gene in 1 ng of DNA, based on Qubit quantification, according to the manufacturer’s protocol. Fragment analysis was carried out on SeqStudio (Thermo Fisher Scientific, Waltham, MA, USA). DNA profiling was achieved through the Microsatellite Analysis software on Thermo Fisher Cloud. 

### 4.6. Mitochondrial DNA (mtDNA) Sequencing

mtDNA characterization was performed using a BigDye Direct Cycle sequencing kit (Thermo Fisher Scientific, Waltham, MA, USA), according to the manufacturer’s protocol. Hypervariable Region 1 (HV1), between 16020 and 16390, and Hypervariable Region 2 (HV2), between 60 and 320, were sequenced on SeqStudio (ThermoFisher Scientific, Waltham, MA, USA). Using the BioEdit program (Ibis Bioscience, Carlsbad, CA, USA) and Sequencher 5.4.6. (Gene Codes Corporation), sequence alignment with the revised Cambridge Reference Sequence was achieved. A haplogroup assignment was carried out using the EMPOP mtDNA database, v4/R13 (https://empop.online/, accessed on 25 August 2022). 

### 4.7. Statistical Analysis

Statistical analyses were carried out in SPSS^®^ Statistics version 26 (IBM). The verification of the data normality hypothesis was carried out by using Cramer–Von Mises Test and Q–Q plots. The variance homogeneity hypothesis was verified using Levene’s test. When the normality hypothesis did not pass, a parametric distribution-free test was applied—specifically, the Kruskal–Wallis test. When normality and variance homogeneity hypothesis passed, a one-way ANOVA was performed. In order to assess the impact of the two factors (swab and fabric) on the different parameters, two-way ANOVA was carried out. 

## 5. Conclusions

The impact of swab types on body fluid identification, applying immunochromatographic tests, is demonstrated for the first time, also ratifying previous studies related to the influence of swab types on nuclear DNA isolation and characterization. Depending on the type of textiles, the use of one swab or another will allow more material to be extracted and tested, stored and/or manipulated for additional analyses. It was also demonstrated that rapid chromatographic tests can perform a dual function: identify body fluids and preserve the remaining sample, obtaining full DNA profiles. This work could also have applications to other currently used immunochromatography tests, such as COVID-19 rapid tests, thus, promoting further research in this area. 

## Figures and Tables

**Table 1:** Study results summary. Rank—the values of band intensity of immunochromatographic test based on a previous scale; ng/μL, DNA concentrations per fabric, and swab type. Replicates are from the same samples in three different experiments.

Fabric	Swab Type	Replicate	Rank	Qubit Quantification ng/μl	Human Quantification ng/μl
		1	G3.5	0.204	0.2319
	Cotton	2	G3	0.159	0.0832
		3	G3.5	0.058	0.015
		1	G7	0.334	0.3666
Denim	4N6FLOQ Swabs Regular	2	G8	0.769	0.6067
		3	G8	1.010	0.8106
		1	G5	1.660	1.5858
	4N6FLOQ Swabs Subungual	2	G7	0.962	0.8649
		3	G8	1.570	1.5468
		1	G5	0.268	0.0785
	Cotton	2	G6	0.223	0.1285
		3	G4	0.273	0.1098
		1	G10	0.896	0.682
Cotton	4N6FLOQ Swabs Regular	2	G9	1.170	0.9544
		3	G10	1.190	1.0185
		1	G7	0.654	0.5249
	4N6FLOQ Swabs Subungual	2	G8	0.378	0.3397
		3	G5	0.397	0.3071
		1	G6	0.233	0.1148
	Cotton	2	G7	0.253	0.0856
		3	G8	0.470	0.1788
		1	G9	0.218	0.0022
Polyester	4N6FLOQ Swabs Regular	2	G7	0.232	0.2105
		3	G7	0.648	0.4905
		1	G5	0.270	0.2401
	4N6FLOQ Swabs Subungual	2	G6	0.192	0.1582
		3	G7	0.748	0.7569

## Data Availability

The data presented in this study are available upon request from the corresponding author. The data are not publicly available for privacy.

## Supplementary Materials

The following supporting information can be downloaded at: https://www.mdpi.com/article/10.3390/ijms231810686/s1.
