# Supplementary material for: The Perfect Match: Assessment of Sample Collection Efficiency for Immunological and Molecular Findings in Different Types of Fabrics
Source: Int J Mol Sci. 2022 Sep 14;23(18):10686. doi: 10.3390/ijms231810686 (PMC9502974; doi:10.3390/ijms231810686)

Supplementary material: DNA Quality Profile Boxplots (RFU)

Denim Cotton Swabs

Total Peak Height

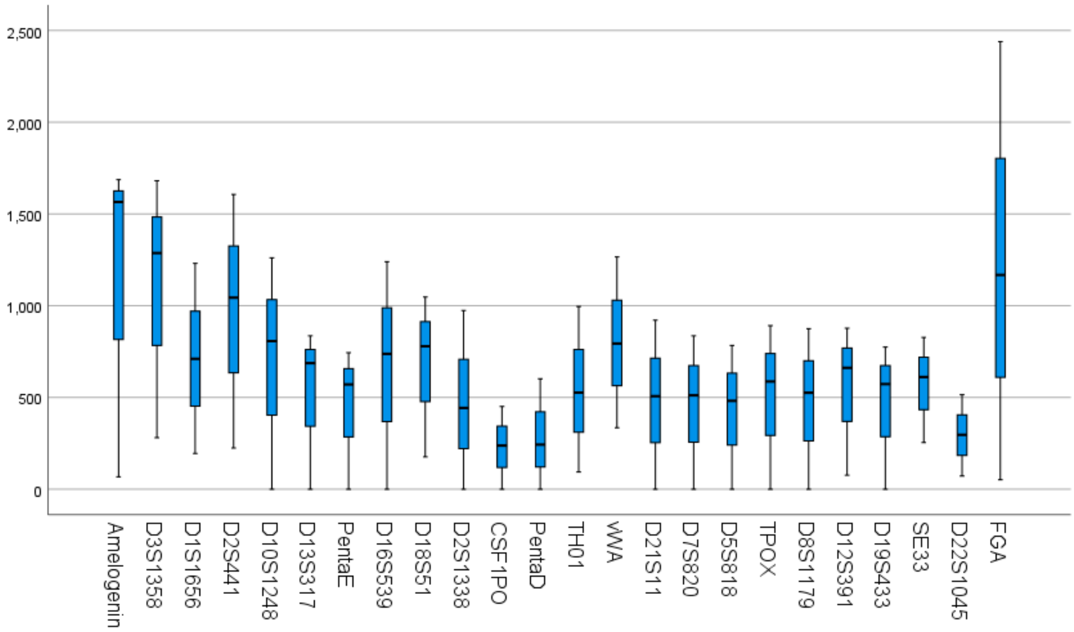

Peak Height Ratio

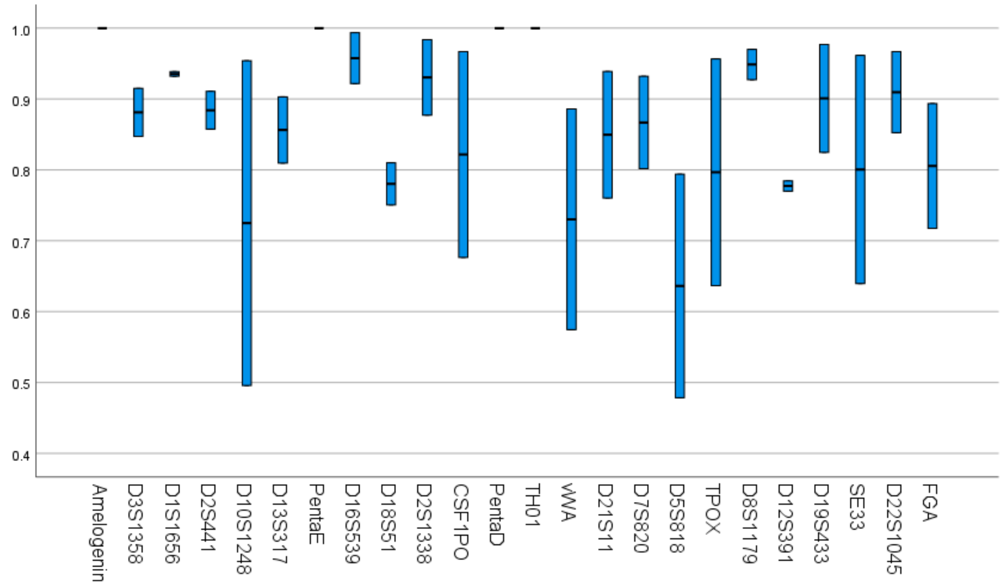

Interlocus Balance

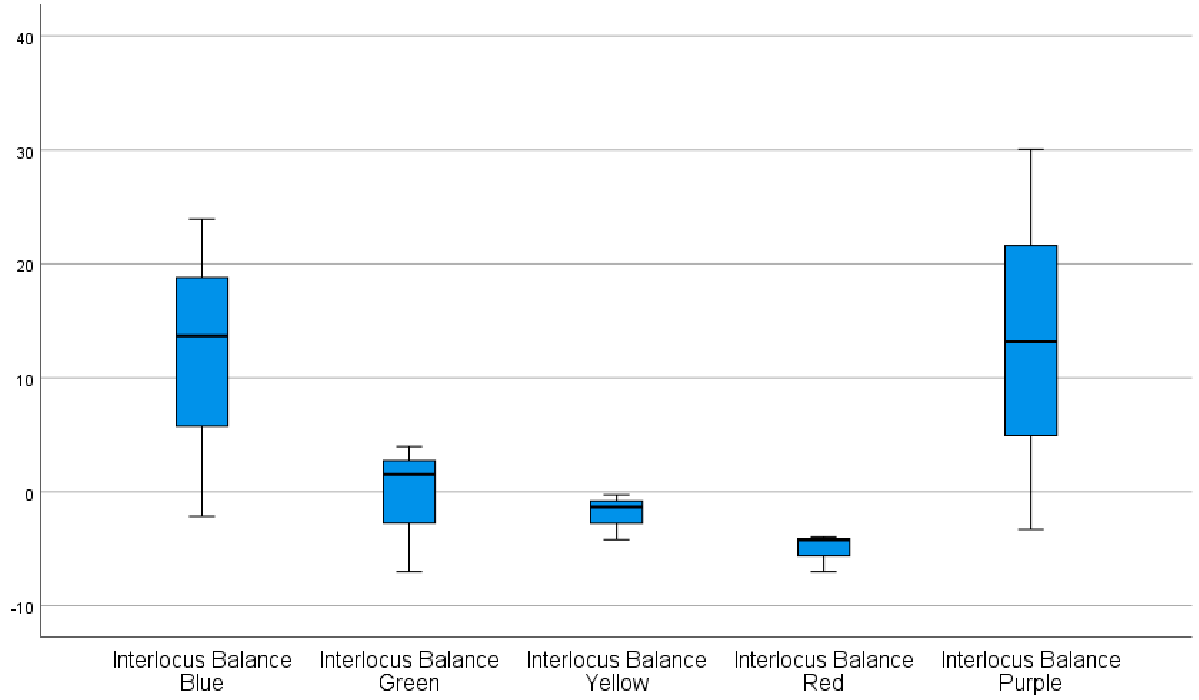

Denim Flock Swabs

Total Peak Height

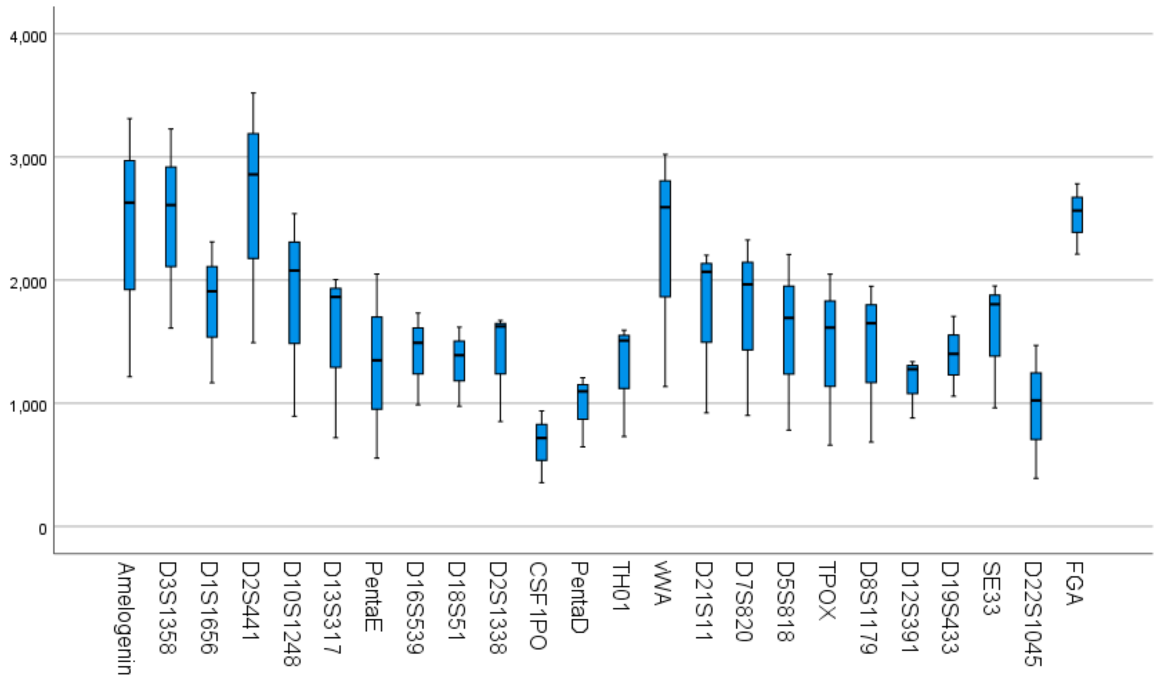

Peak Height Ratio

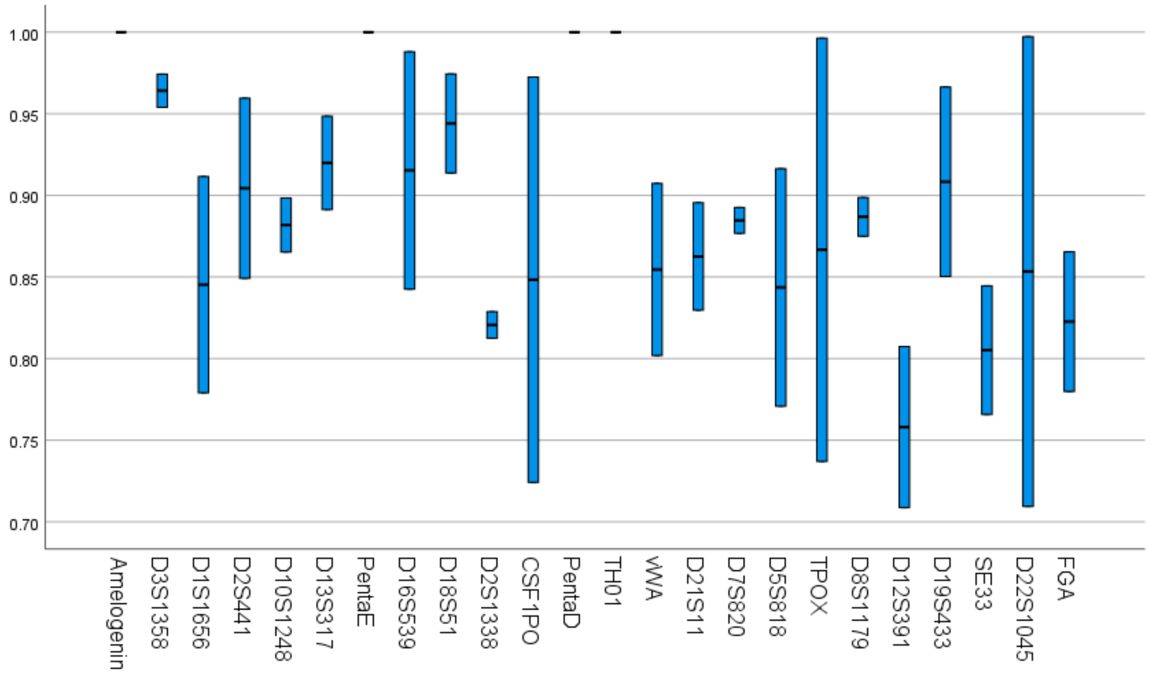

Interlocus Balance

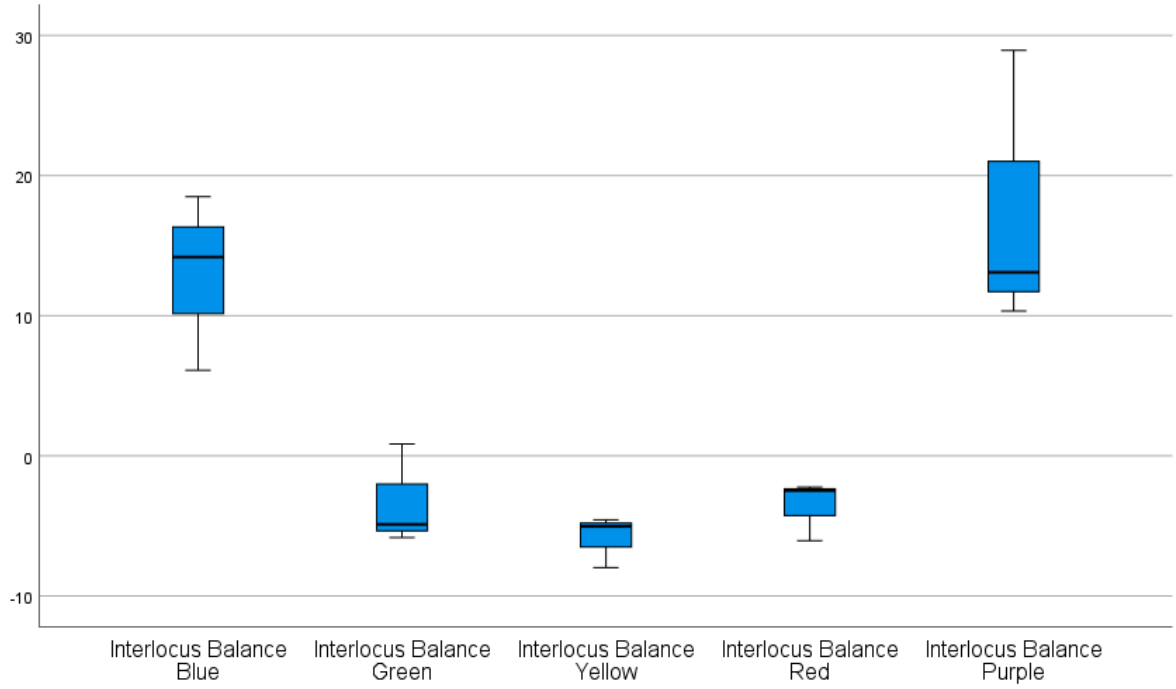

Denim Subungal Swab

Total Peak Height

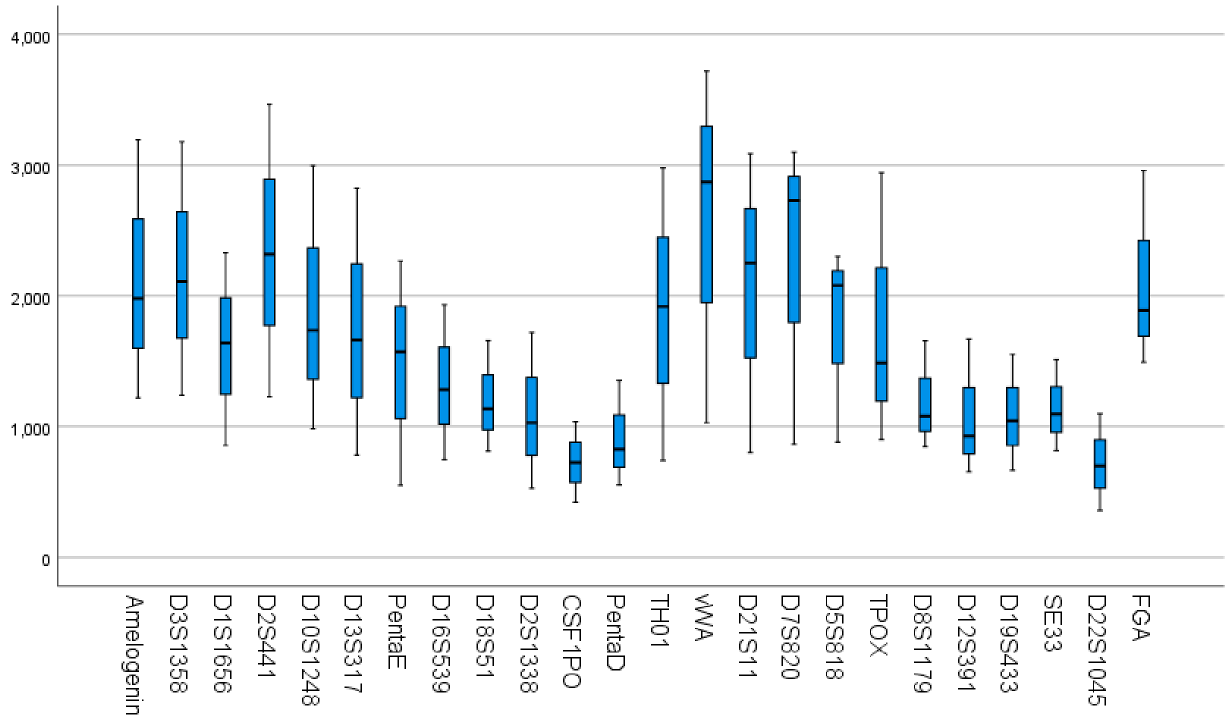

Peak Height Ratio

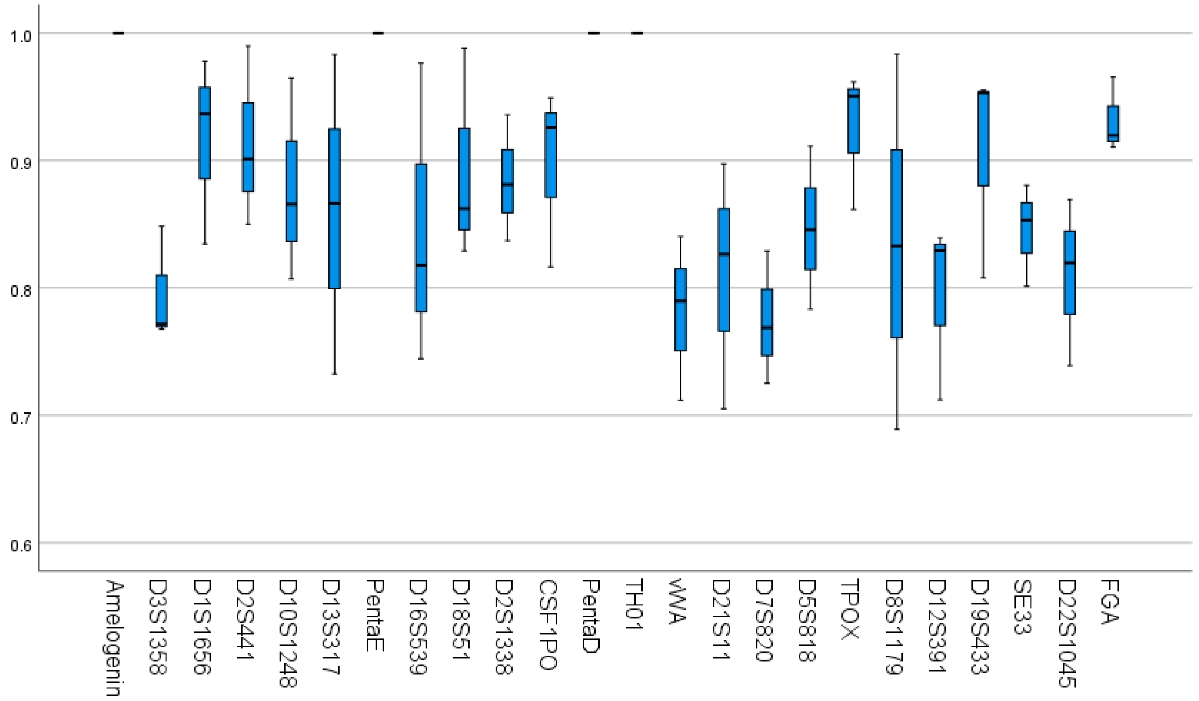

Interlocus Balance

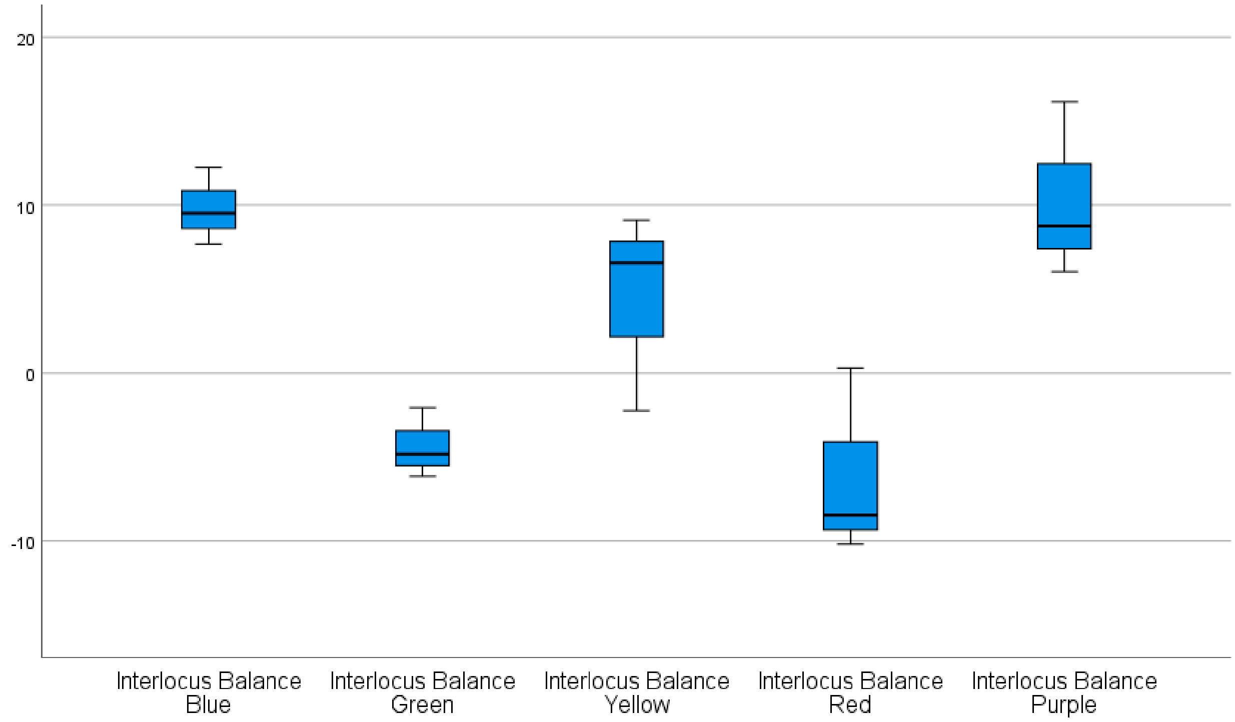

Cotton Cotton Swabs

Total Peak Height

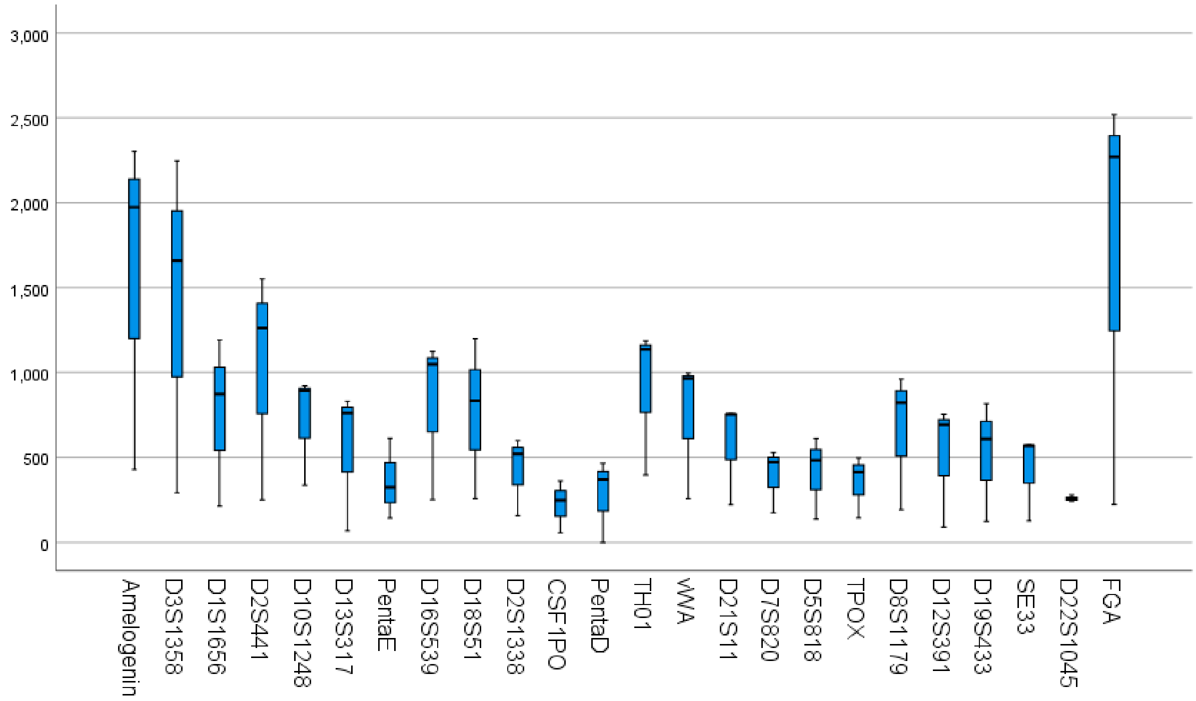

Peak Height Ratio

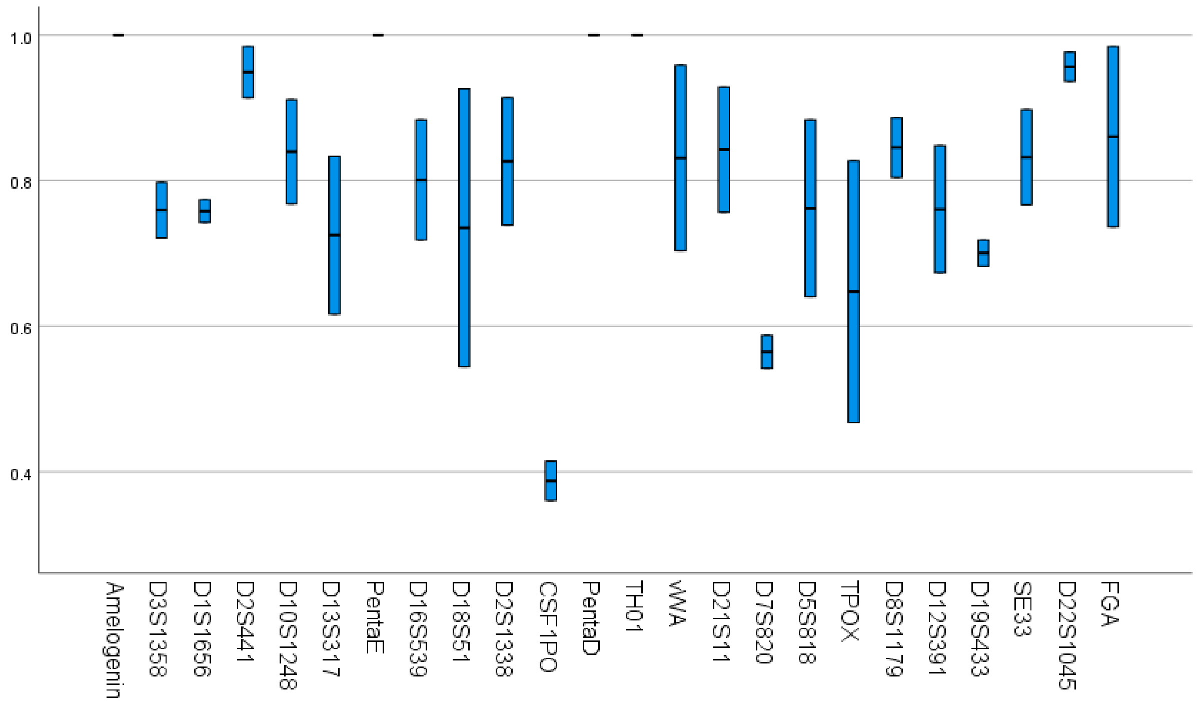

Interlocus Balance

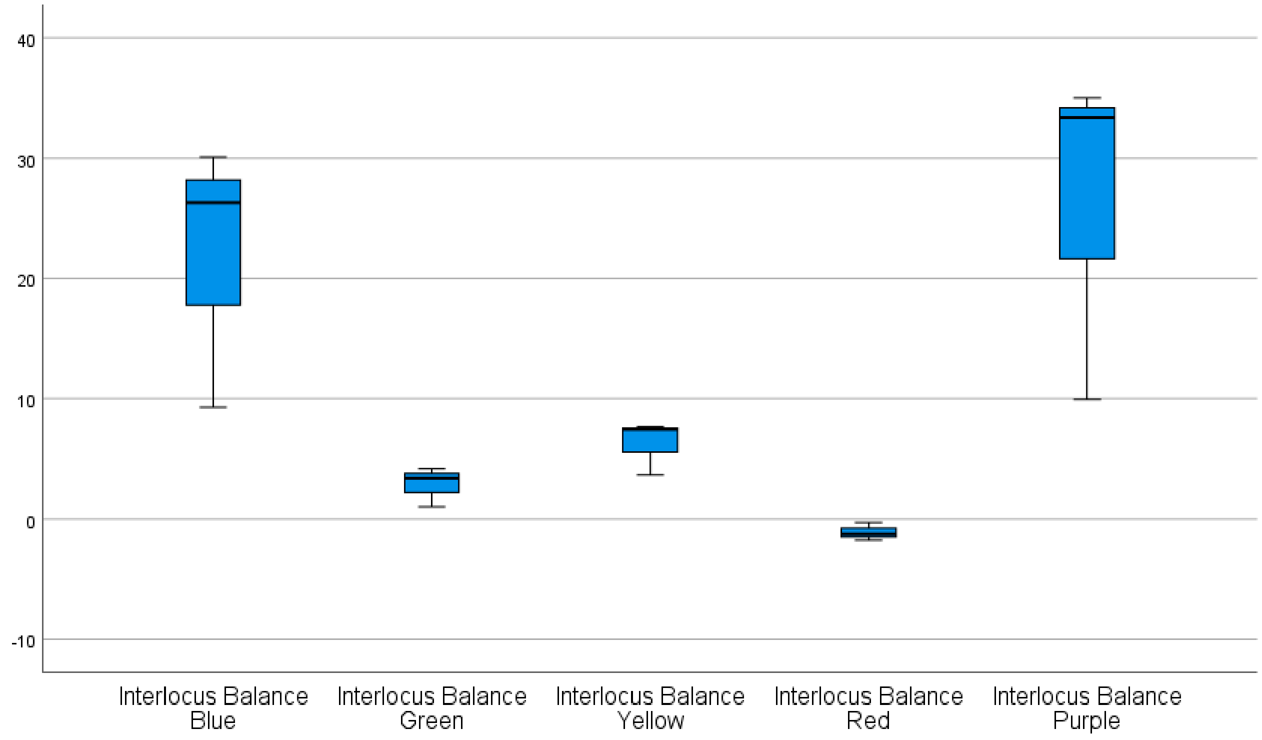

Cotton Flock Swabs

Total Peak Height

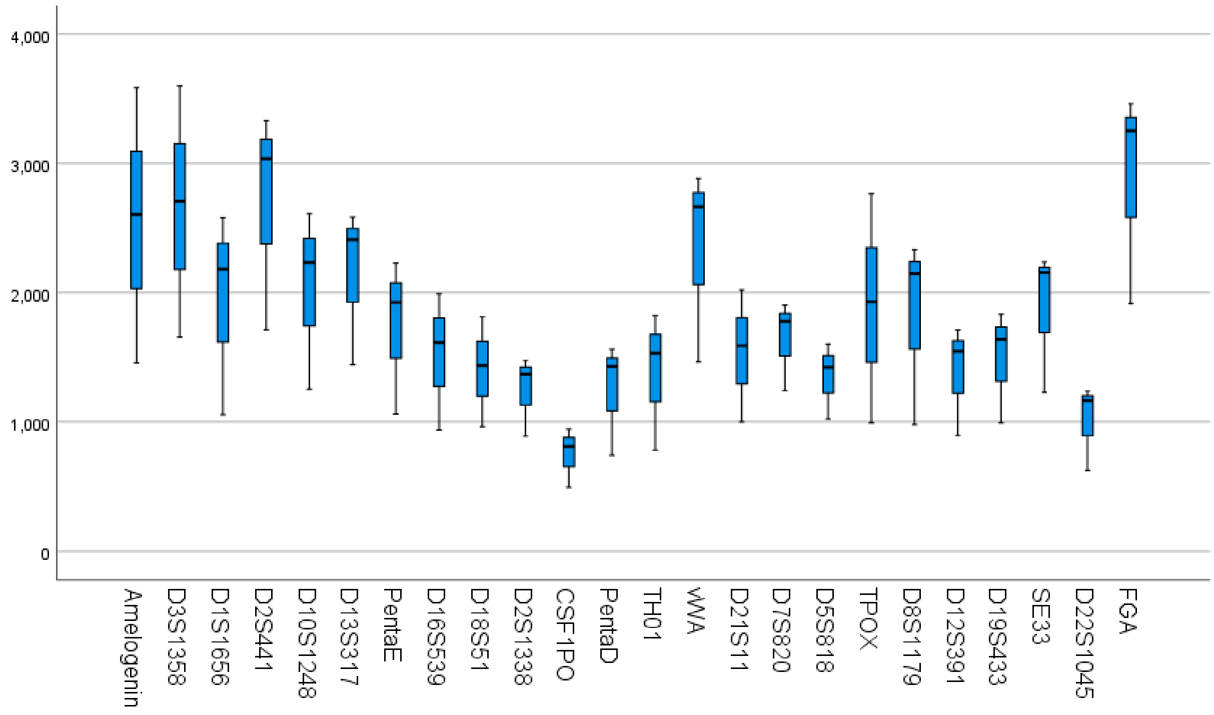

Peak Height Ratio

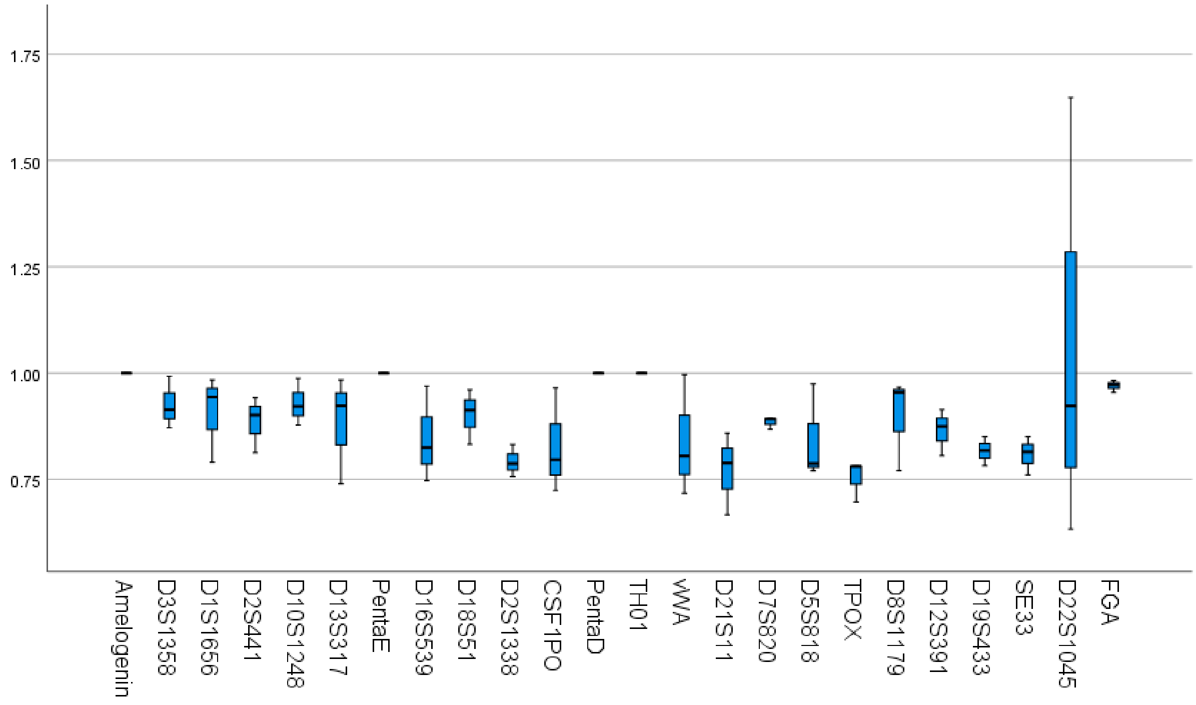

Interlocus Balance

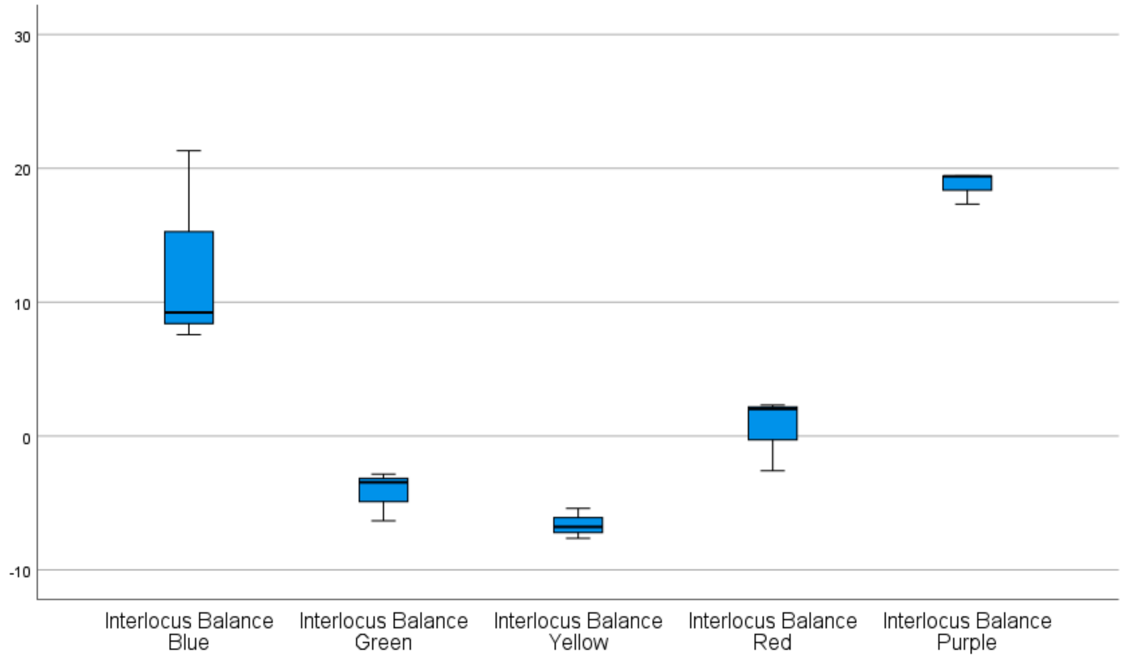

Cotton Subungal Swabs

Total Peak Height

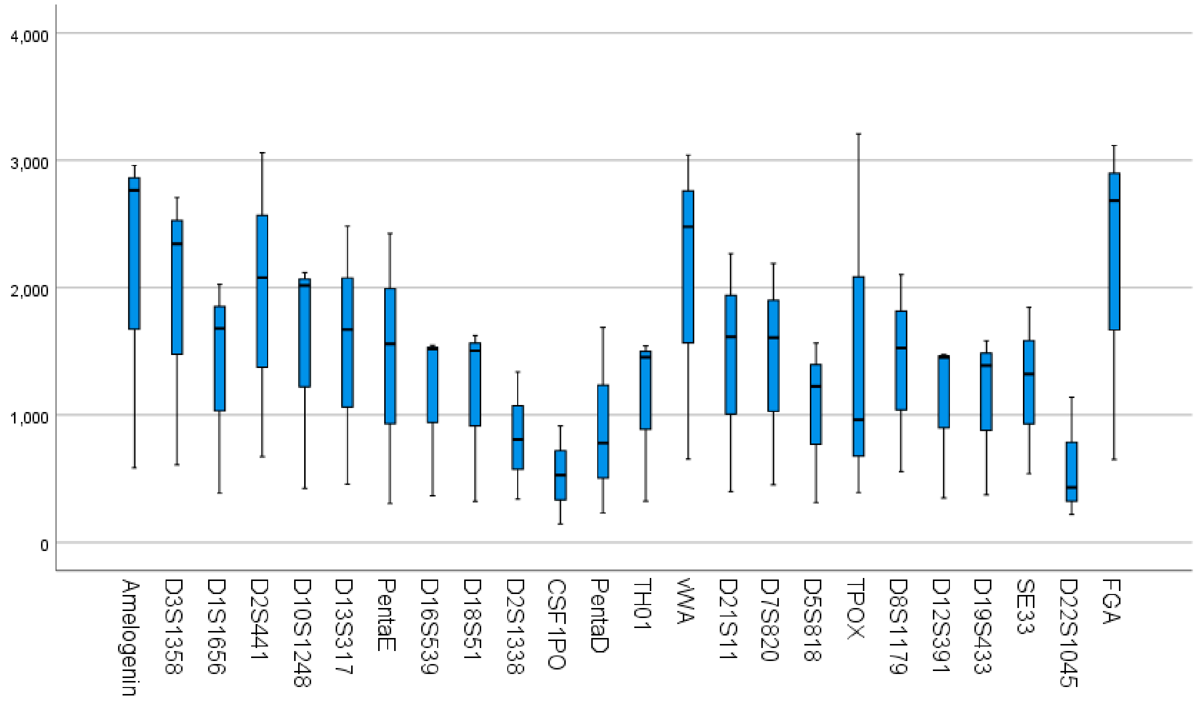

Peak Height Ratio

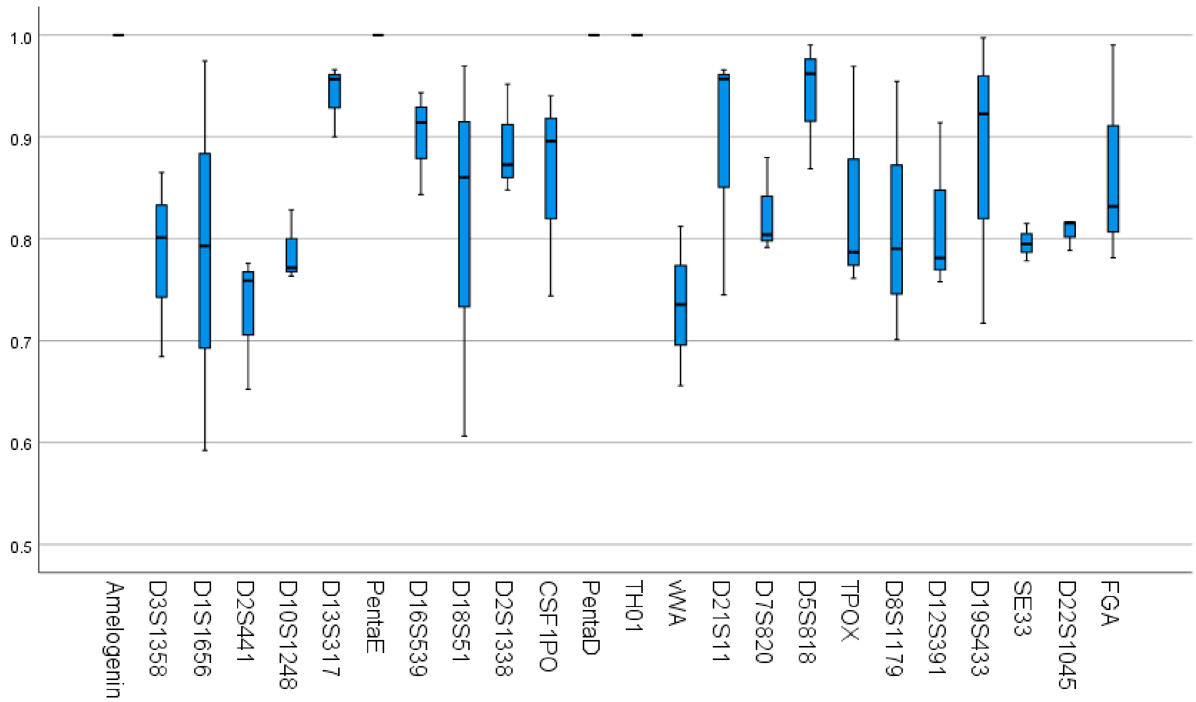

Interlocus Balance

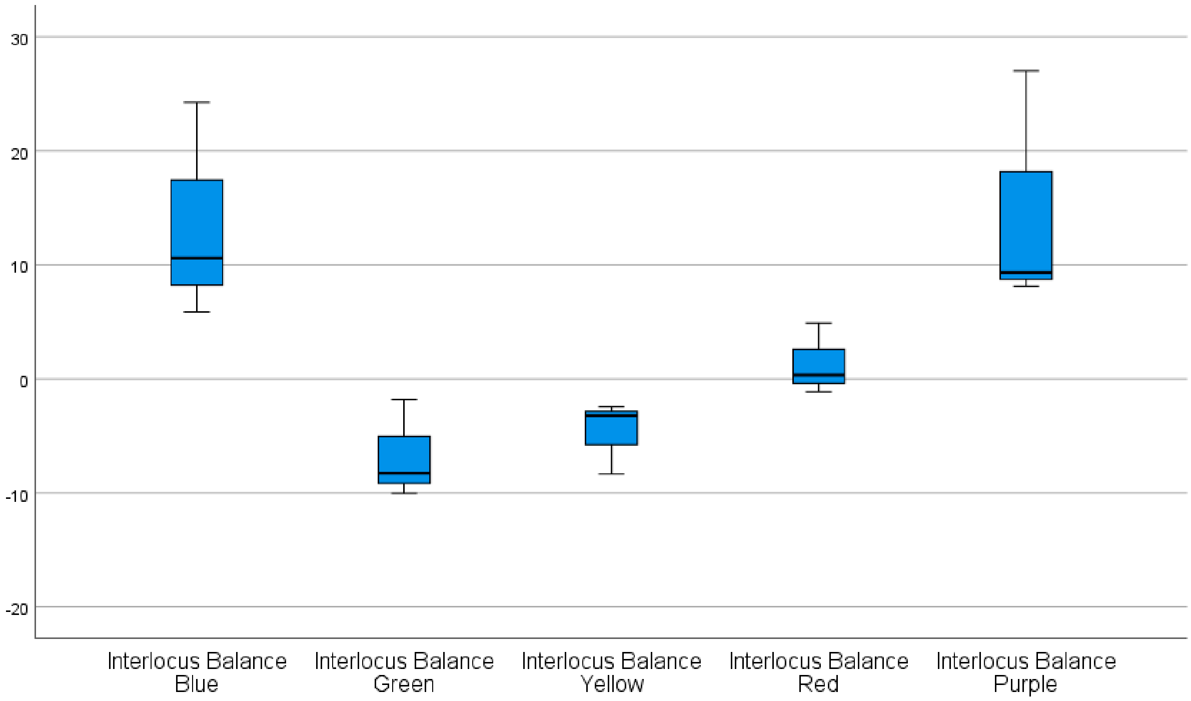

Polyester Cotton Swab

Total Peak Height

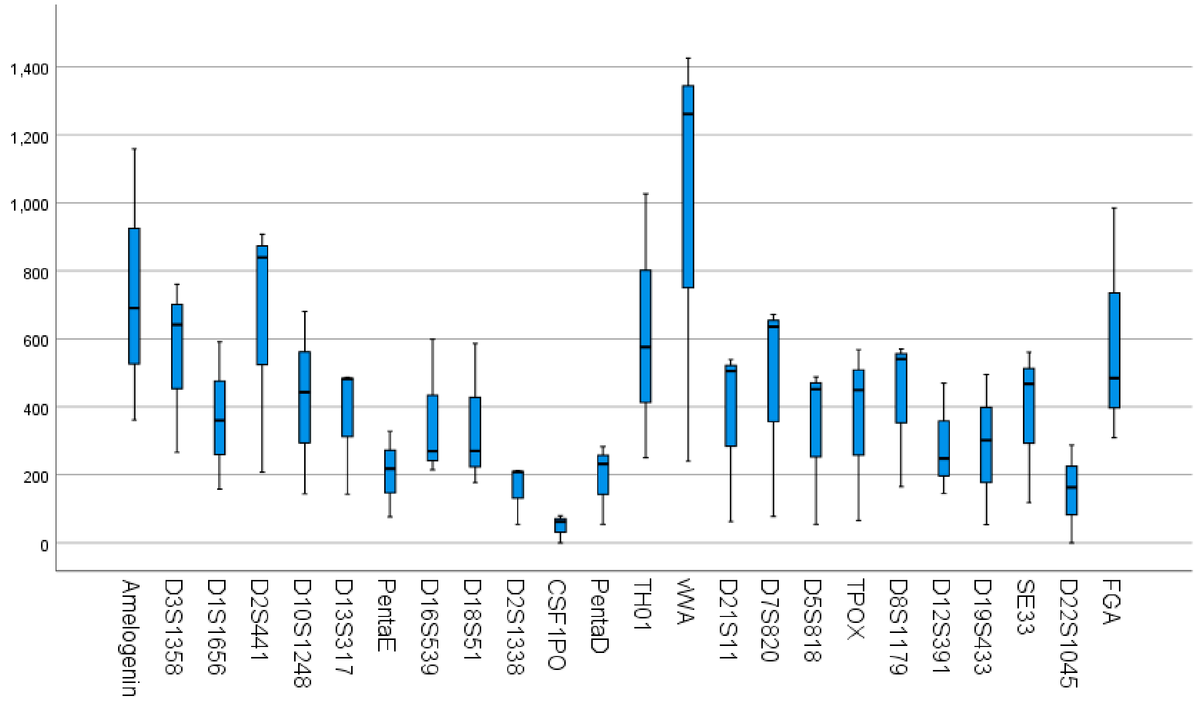

Peak Height Ratio

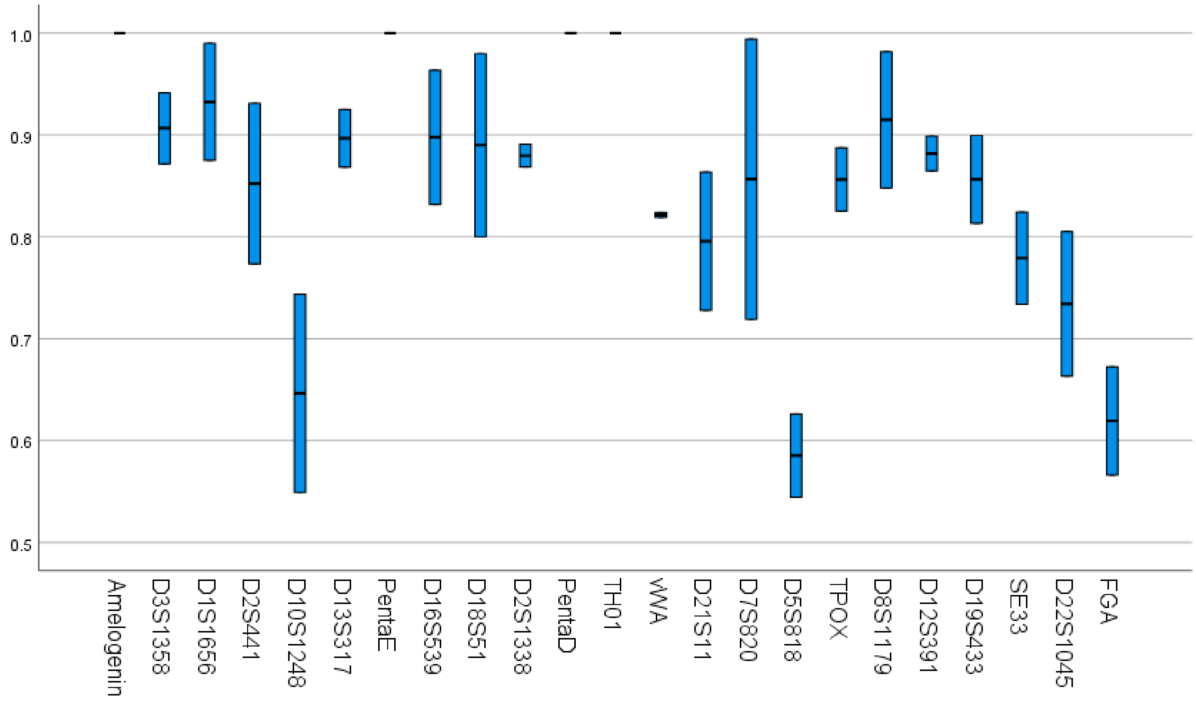

Interlocus Balance

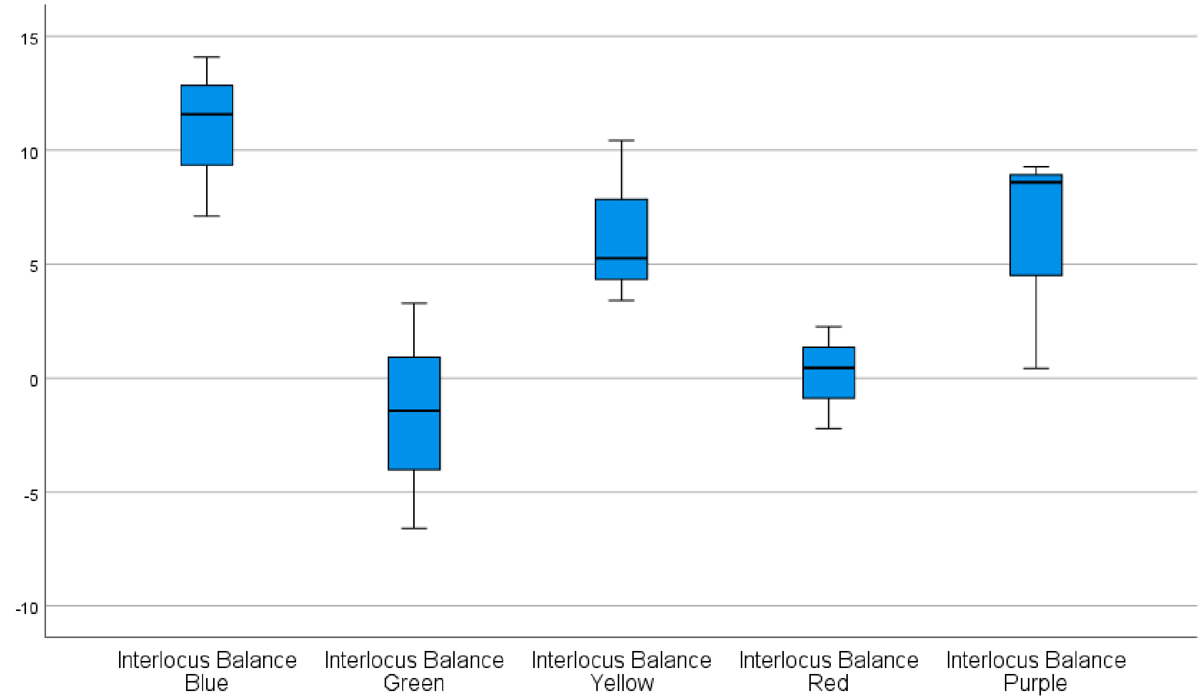

Polyester Flock Swab

Total Peak Height

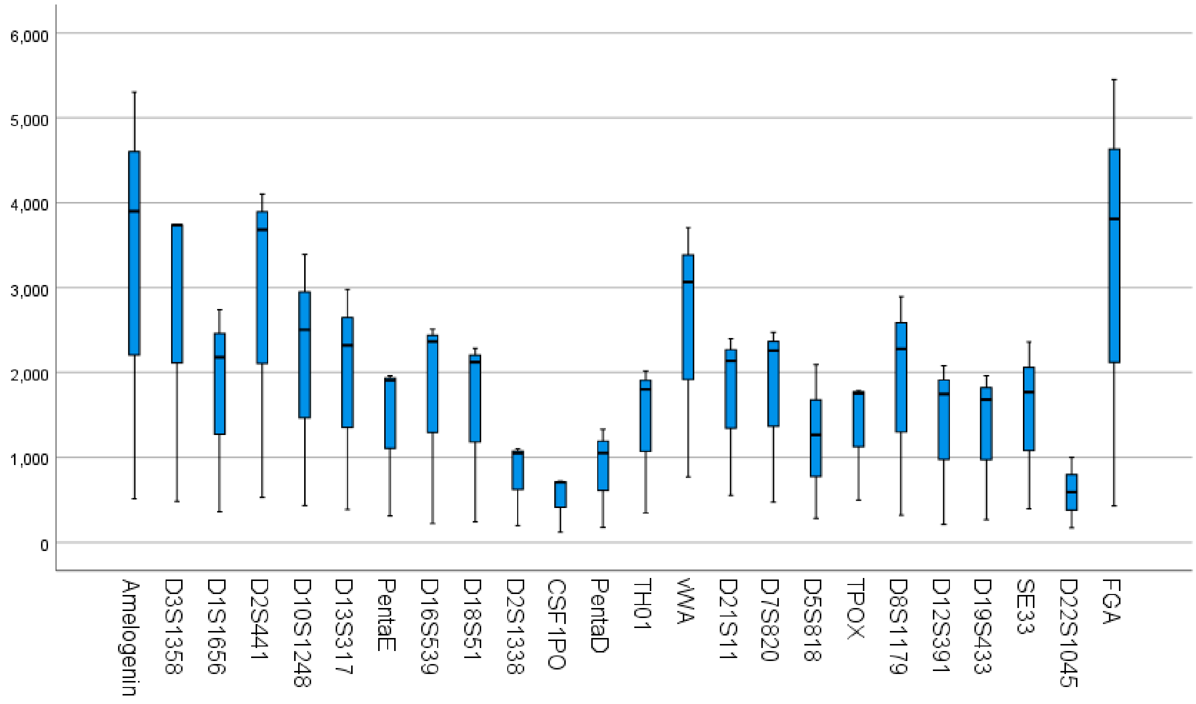

Peak Height Ratio

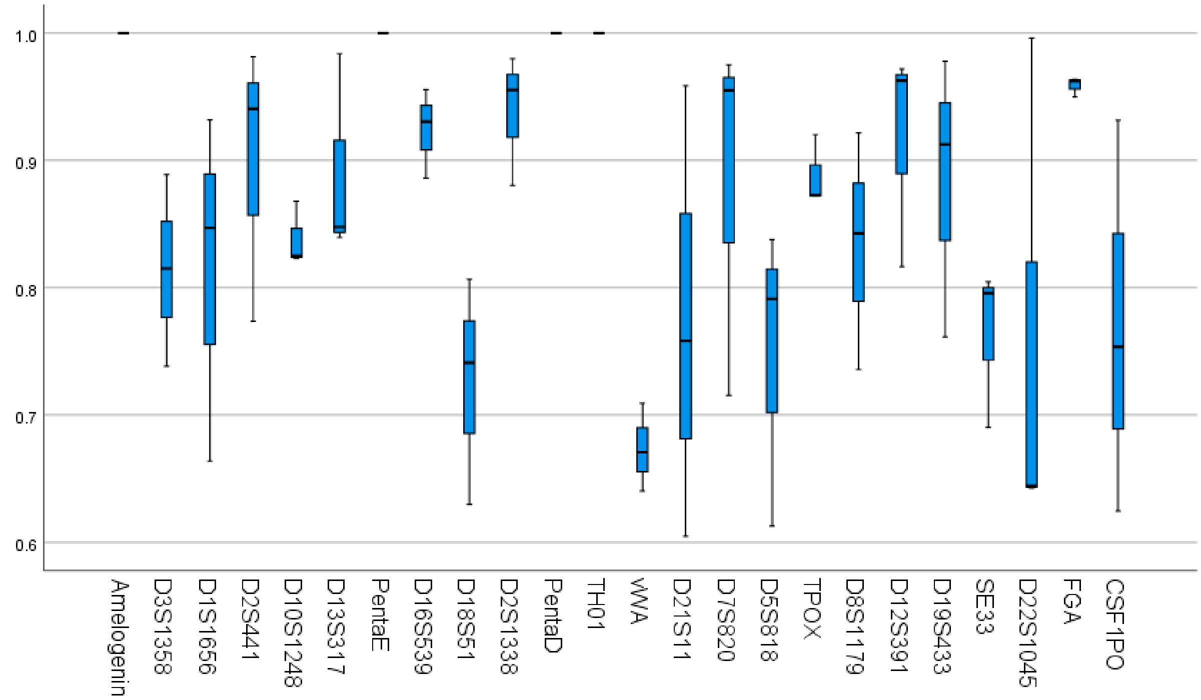

Interlocus Balance

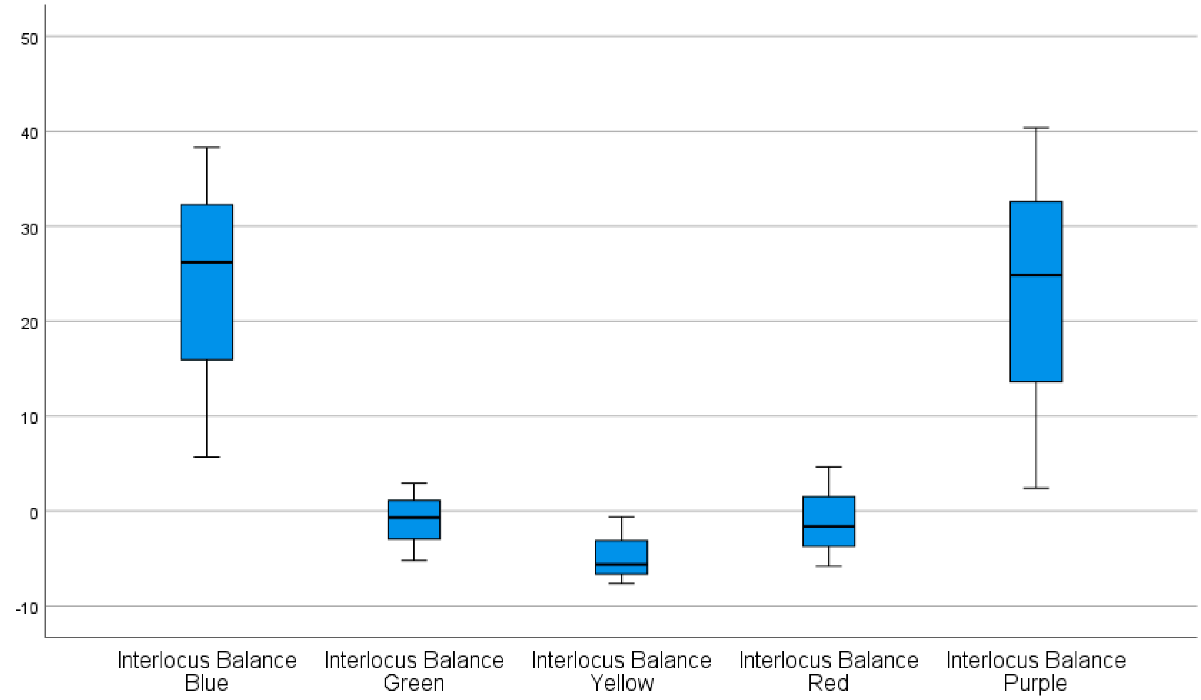

Polyester Subungal Swab

Total Peak Height

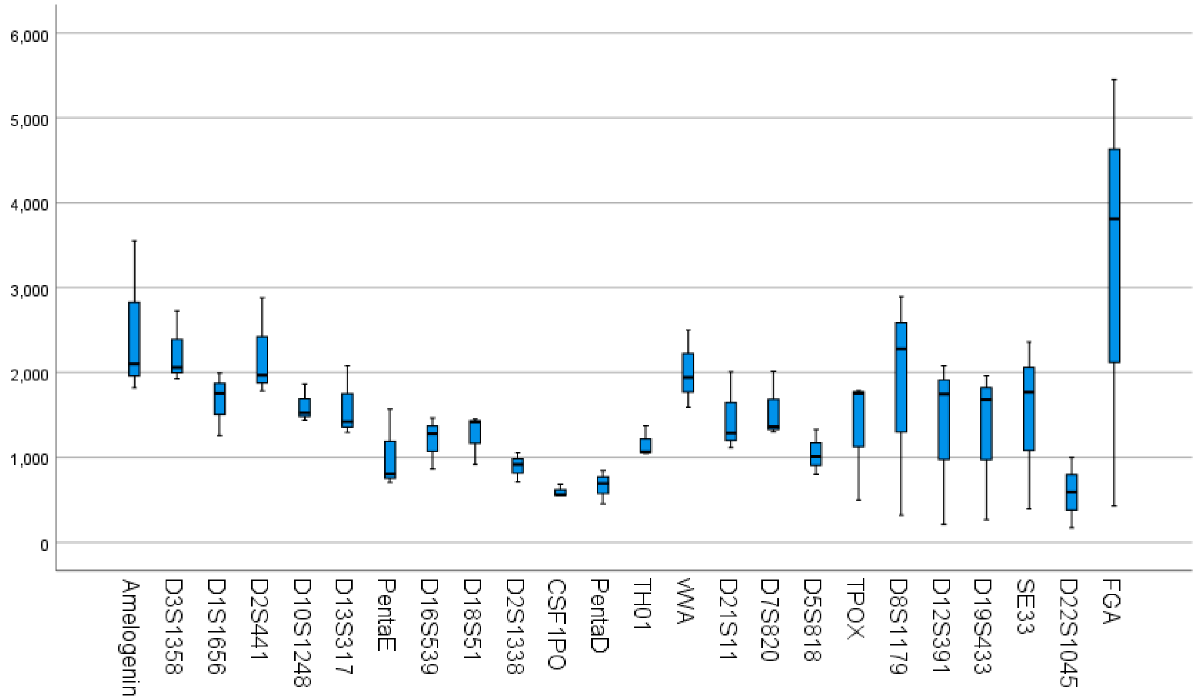

Peak Height Ratio

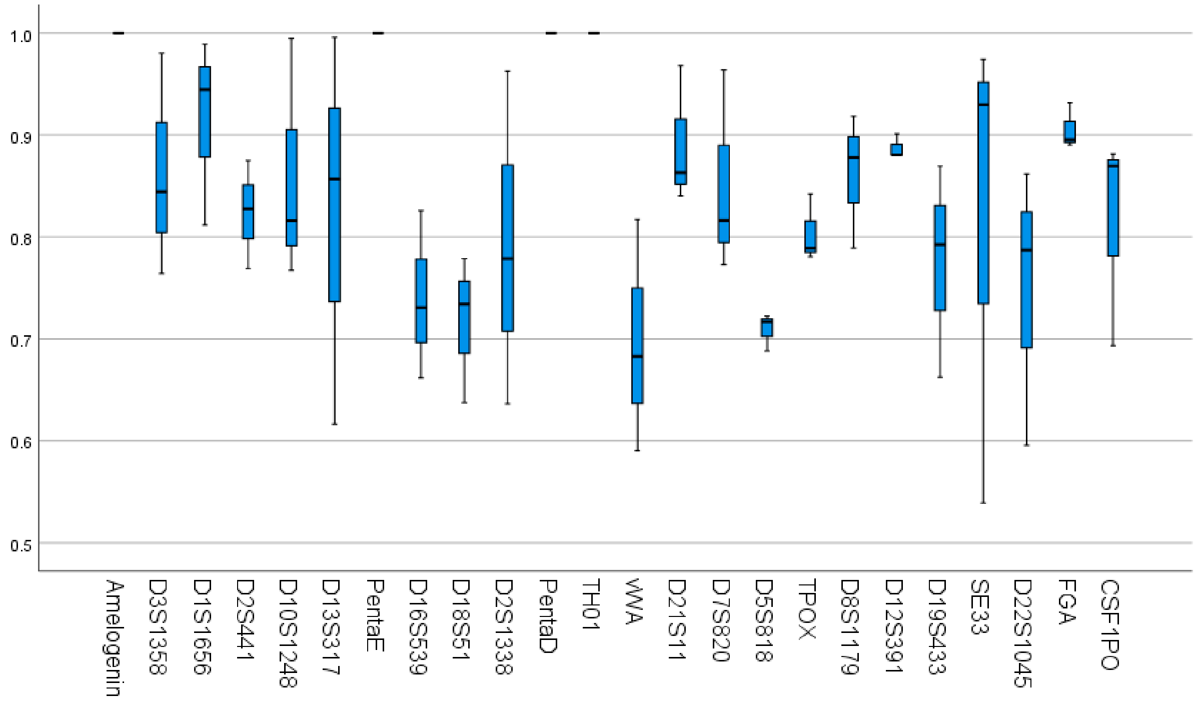

Interlocus Balance

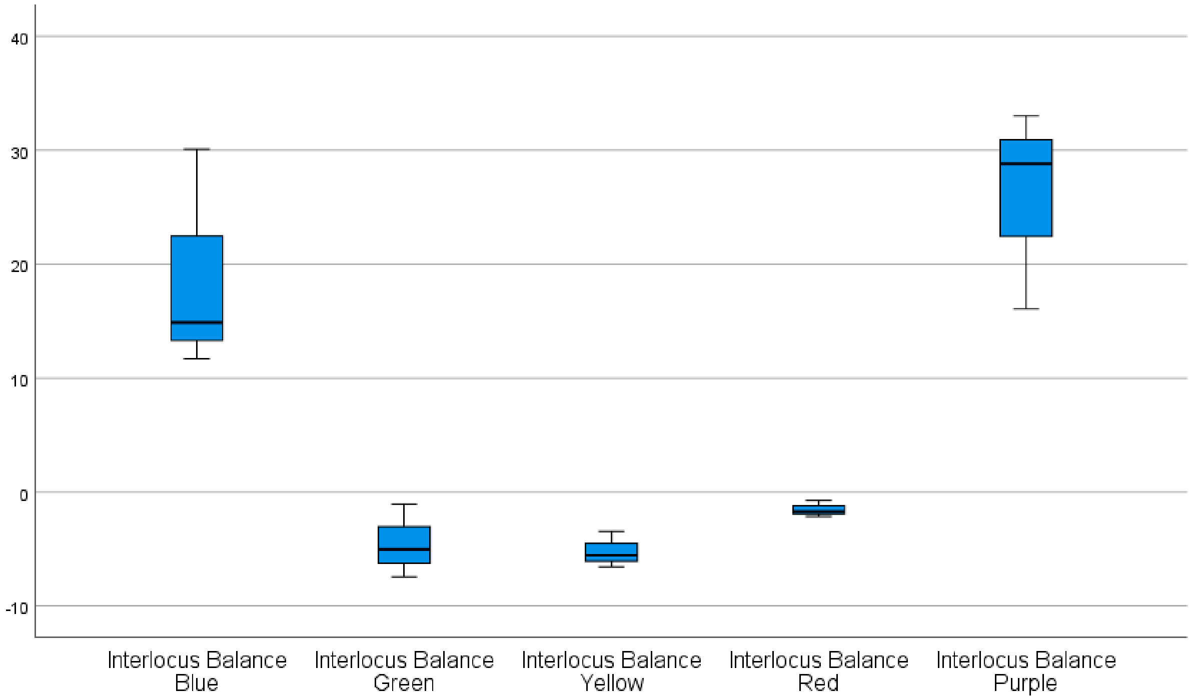

Supplement: Supplementary file 1 [file ijms-23-10686-s001.zip › ijms-1910546-supplementary.pdf]
